# *Shark Dive* and *Hologram Zoo*: Two Case Studies of Virtual Animal Encounters as Possible Models for Sustainable Wildlife Tourism

**DOI:** 10.3390/ani14060926

**Published:** 2024-03-17

**Authors:** Rebecca Scollen, Andrew Mason

**Affiliations:** 1 School of Creative Arts, University of Southern Queensland, Toowoomba, QLD 4350, Australia; 2 School of Humanities and Communication, University of Southern Queensland, Toowoomba, QLD 4350, Australia; andrew.mason@usq.edu.au

**Keywords:** Australia, animals, sharks, virtual encounters, wildlife tourism, zoos

## Abstract

**Simple Summary:**

Wildlife tourism is commonly argued to positively contribute to nature conservation by providing humans with educational and meaningful first-hand experiences with animals. However, concern for the sustainability of nature due to the negative impacts of wildlife tourism on animals and the environment invites alternative virtual opportunities for close encounters. Participant Observation framed by an Animal Studies perspective is employed to interpret two contemporary Australian family entertainments, *Shark Dive* and *Hologram Zoo*, centred on providing people with wild animal interactions but without real animals present. *Shark Dive* is a theatrical puppetry production replicating a real shark dive, and *Hologram Zoo* is an augmented reality experience that displays a variety of animals in 3D. The content of both is examined to understand how the animals are imagined, experienced, and given significance, to determine whether they offer viable alternatives, or complementary additions to in situ wildlife tourism. Findings reveal both hold potential for virtual wildlife tourism. *Shark Dive* provides a positive representation of marine life, generating awareness about conservation and encouraging self-reflection. *Hologram Zoo*’s depiction of wild animals is impressive, but to more closely align with contemporary wildlife tourism ideals, the experience could enhance its conservation messaging and positive human–animal interactions.

**Abstract:**

Wildlife tourism is commonly argued to positively contribute to nature conservation by providing humans with educational and meaningful first-hand experiences with animals. However, concern for the sustainability of nature due to the negative impacts of wildlife tourism on animals and the environment invites alternative virtual opportunities for close encounters. Participant Observation framed by an Animal Studies perspective is employed to interpret two contemporary Australian family entertainments, *Shark Dive* and *Hologram Zoo*, centred on providing people with wild animal interactions but without real animals present. *Shark Dive* is a theatrical puppetry production replicating a real shark dive, and *Hologram Zoo* is an augmented reality experience that displays a variety of animals in 3D. The content of both is examined to understand how the animals are imagined, experienced, and given significance to determine whether they offer viable alternatives or complementary additions to in situ wildlife tourism. Findings reveal both hold potential for virtual wildlife tourism. *Shark Dive* provides a positive representation of marine life, generating awareness about conservation and encouraging self-reflection. *Hologram Zoo*’s depiction of wild animals is impressive, but to more closely align with contemporary wildlife tourism ideals, the experience could enhance its conservation messaging and positive human–animal interactions.

## 1. Introduction

Wildlife tourism is argued to positively contribute to nature conservation by providing meaningful first-hand experiences with wildlife [1,2,3]. Enjoyable, educational, and emotional encounters can increase tourists’ understanding of the animals observed and of the environmental issues that impact them [4,5]. However, wildlife tourism can have short-term impacts on wildlife, which in turn, may have long-term consequences in their lives [6]. Impacts caused by human activity include habitat degradation, the introduction of disease, change in animal behaviours, and stress, harm, and risk of death to animals [7,8]. Concern for the sustainability of nature due to wildlife tourism activity invites alternative virtual opportunities for close encounters [4,7]. While they are not promoted specifically as wildlife tourism experiences, this paper presents two contemporary Australian family entertainments, *Shark Dive* and *Hologram Zoo.* Both are centred on providing people with wild animal interactions but without real animals present. The aim of this research is to observe and detail the ways in which the animals are represented and displayed for human consumption in *Shark Dive* and *Hologram Zoo*. This investigation is prompted by the inquiry: do *Shark Dive* and *Hologram Zoo* offer viable alternatives, or complementary additions, to in situ wildlife tourism offerings? Through the direct experience of these virtual presentations, is it evident that they aid conservation and contribute to the improvement of human interactions with animals and nature, thus reinforcing sustainable wildlife tourism ideals? The following sections provide context for *Shark Dive* and *Hologram Zoo* by highlighting the positive and negative impacts of shark diving tourism and zoo tourism, which the two family entertainments represent virtually and respectively. This then prompts the final section, which discusses virtual animal encounters as another possible way to engage humans with other species.

### 1.1. Shark Diving Tourism

Shark diving tourism is a thriving international industry with more than 20 countries [9] offering the chance to experience sharks up close in their own natural environments [5,6]. In Australia, wildlife tourists can snorkel amongst whale sharks and scuba dive with reef sharks and grey nurse sharks at dedicated sites in the western and eastern coastal waters of the country, respectively [10]. White shark cage-diving occurs at the Neptune Islands off the coast of South Australia and has been offered as a tourism experience since the 1970s [5]. Australia was the first to engage people in this particular kind of shark-dive activity [1], where divers are housed inside a ‘surface cage’, which white sharks approach mostly due to the bait used to lure them [9]. Cage-diving is a popular activity because it provides a thrilling up-close encounter, enhancing participants’ excitement and sense of awe of the apex predators [3,11]. Cage-diving enables participants to observe the white sharks in their natural habitat, increasing their awareness of the species and the enjoyment of the perceived naturalness of the encounter [5]. This can increase their sense of connection with nature and their intentions to adopt conservation behaviours [12]. For example, a study of tourists found white shark cage-diving increased participants’ understanding, awareness, and concern for sharks and their conservation; general interest in sharks; and conservation-related behaviours (e.g., talking positively about sharks to others via social media) [3].

However, there are a range of concerns about the impacts of cage-diving practices on marine life and shark-human relations. Some examples in the Neptune Islands include: an increase in large numbers of schooling fish due to regular use of bait [9]; a change in habitat use and residency of smooth stingrays [13]; the encouragement of white sharks to remain at the surface of the ocean to follow baited lines, whereas they typically stalk their prey from below [11]; and the possibility of white sharks being distracted from normal foraging behaviours in favour of perceived opportunities to obtain bait [9]. This wildlife tourism activity also raises safety concerns for humans due to fears that white sharks are essentially becoming ‘trained’ to approach boats and people, which may increase the likelihood of contact with others beyond the tourism experience [11]. However, cage-diving advocates argue that as long as the practice is responsibly performed, the benefits outweigh the concerns as it provides an enjoyable experience that debunks negative myths about sharks and encourages conservation efforts [11].

### 1.2. Zoo Tourism

Per year, approximately 700 million people visit accredited zoos and aquaria worldwide and spend, on average, four hours onsite [14,15]. Zoo tourism is a popular family entertainment, bringing people in close contact with an array of wild but confined animals; reportedly, 100 million of them held captive internationally [16]. There is a long history of zoological institutions welcoming the public to “view the diverse, exotic and captivating animal kingdom with whom we share our planet” [16] (p. 61). Modern zoos have entertained visitors since the late 1700s, originally derived from private menageries [17], then expanding throughout the 1800s to become public institutions designed for conserving and scientifically studying endangered species [18]. By the mid-20th century, significant public concerns pertaining to the poor treatment and living conditions for wild animals placed great pressure on zoos to adjust practices and infrastructure in order to sustain themselves [16]. At this time, many repositioned themselves as places of free-choice learning and conservation education [12,19]. The focus on conservation and the responsibility of zoos to raise awareness, connect people to nature, and encourage sustainable behaviours [20,21] has continued into the 21st century, with some promoting themselves as a safe refuge for injured animals [22] as part of this overall conservation objective. As part of the conservation education commitment by zoos, onsite interpretation is employed to inform visitors about “animals, habitats and conservation issues” [12] (p. 182) with the view to “connecting (humans) emotionally and cognitively to animals” [15] (p. 64). Increased understanding and advocacy are thought possible when there is plenty of information provided by onsite interpretation [15], along with time for self-reflection included in the experience [3].

Although it is noted that many zoos have significantly improved over the last 50 years when it comes to animal welfare and to conservation education, concerns about zoos’ ethical appropriateness and their sustainability as tourism organisations remain rife. Fennell [8] (p. 91) likens zoos to prisons “where artificial space is created to impose occupancy and... demonstration, (which) violates animals’ status as free-living creatures...”, leading to psychological stress and poor health as their ecological needs are unmet [8] (p. 84). The keeping of animals in unnatural settings and enclosures curtails normal behaviour, and as greater understanding of individual species grows, so too does awareness of their suffering in captivity [16] (p. 61). Zoo visitors’ presence, along with their direct interaction with animals, can create fear and stress leading to behaviour changes, such as avoidance, hiding, aggression, and reduction in foraging, grooming, and play [23] (p. 8-9). The viability of zoos as breeding centres to reintroduce animals to the wild is questioned because they are seen to be breeding “generations of captive animals who are used to humans” [16] (p. 72) and not accustomed to living in their natural environments. Although marketing themselves as conservation educators, Burns and Benz-Schwarzburg [2] (p. 42) argue that “zoos rarely provide *in-depth* information about conservation” for their visitors. Finally, research undertaken by Packer et al. [20] (p. 98) suggests that a large proportion of visitors attracted to zoos hold values (e.g., self-interest) that are not aligned with a conservation agenda, and so, the pro-environment messaging from zoos and their staff is not resonating as effectively as hoped with many visitors, and in some cases is understood to be an “attack on their values”. This finding confirms that for most people visiting the zoo is an enjoyable wildlife tourism experience consumed for entertainment and for seeing animals up close [16,24], rather than for reaffirming or supporting conservation ideals.

### 1.3. Virtual Animal Encounters

Human encounters with animals can bring us joy and a sense of connection with other species on Earth. As discussed, there are a range of benefits from such experiences, but also risks and negative impacts. Concern for the sustainability of nature due to wildlife tourism activity invites alternative opportunities for close encounters [4,7]. These can also potentially foster interest and understanding in animals, as well as deepen our relationships with them and with the natural environment. Von Essen et al. [25] (p. 8) pose the question ”to what extent fully or semi-virtual animal-based tourism may replace or complement ‘real’ encounters, thus taking some pressure and stress off the animals in their habitats, enclosed or wild?”. Virtual animal encounters are wide-ranging, with some having been in existence for hundreds of years via puppetry [2], live theatre [26], and film [27], through to more recent times employing new technologies such as animatronics [28], drones [25], and computer-generated experiences via virtual and augmented realities [29] and gaming [30]. The virtual nature of these experiences means that real animals are not usually directly involved, thus, not intruded upon by human demands for their presence and service.

Although not physically present, audiences can vicariously experience nature [31] via mediated presentations to gain insights into flora and fauna. The portrayal of animals and the environment shapes audience understanding and perceptions [30,32], and so, it is important that these virtual representations are designed and created thoughtfully. Our learned perspectives about animals and our relations with them can affect the way we treat them, which, if based on anthropomorphised, anthropocentric, and inauthentic virtual depictions, is likely to be as commodities without agency, furthering harm [7] (pp. 11–12). However, if created responsibly, virtual encounters have been shown to “generate positive and powerful responses amongst audiences” [4] (p. 757). People can become immersed in a virtual experience, feel emotionally connected to nature, and indicate a willingness to change environmental behaviours [4]. Importantly, Hofman et al. [4] (p. 757) demonstrate that whether engaged in a virtual experience or a real experience (of the Great Barrier Reef in this instance), the level of engagement with the reef and the increase in pro-conservation commitment to it is the same.

The Great Barrier Reef virtual experience is an example of an encounter derived by virtual reality technology, where the “user is fully immersed into a virtual environment...resulting in real-time simulation of one or more of the user’s five senses” [29] (pp. 2057–2058). Increasingly, this technology is being employed in the education, gaming, and tourism sectors due to its ability to enhance learning, facilitate interactivity, and generate vicarious experiences [29,30]. Augmented Reality (AR), on the other hand, “…enhances real-world environments using layers of computer-generated images through a device...With AR, a large majority of what the user sees is still real world” [29] (p. 2058). Examples include the German Circus Roncalli [7], where holographic images of animals are displayed inside a real circus tent alongside real human circus performers, and museums and art galleries employing an AR app to operate as a mobile location guide and information dissemination tool [29].

Although using old technology, the incorporation of puppetry to represent animals in live theatre has seen a resurgence over the last couple of decades, with productions such as *The Lion King*, *War Horse*, *Life of Pi*, and *Storm Boy* popular with children and adults alike. Representations of animals in theatrical performances often stand in as “symbols of human behaviour and allegories of human preoccupations” [26] (p. 3). For example, Joey the war horse “is an agent of, and metaphor for, human beings” [33] (p. 537). Yet in some cases, including *War Horse*, the animal is also understood to be the animal s/he is standing in for, generating empathy in the audience and building identification with the relationships presented between humans and animals [33]. Live theatre is an ancient form of storytelling where performers (puppets) and audience members share a dedicated space and time brought together by the narrative. This connection can make theatre feel more immersive than other art forms, with the actors/puppeteers performing their characters live in the space [34]. This, in turn, ensures live theatre is a powerful tool for communication and consideration, engendering empathy and emotional connections that can affect change in audiences and actors [35], including their opinions about socio-political issues [34]. The expertise of puppet designers and puppeteers brings animals to life in productions such as *Life of Pi* by closely replicating the animals’ anatomy, movements, and sounds [36]. Beyond this, it is the audiences’ imagination and willingness to suspend disbelief that ensures the animals and their interaction with each other and with humans is considered authentic.

As puppeteer Caldwell remarks, (it is) “...the offer that you make to the audience: ‘Here’s a tiger. Do you want to agree that it’s real with us?’ That means that they (the audience) then take part in the creation. Intellectually, we know it’s a puppet. But really quickly most people want to buy into the game” [36].

## 2. Materials and Methods

Participant Observation framed by an Animal Studies perspective is employed to interpret two contemporary family entertainments, *Shark Dive* and *Hologram Zoo.* Both creatively replicate either shark diving or zoo attendance with the aim of providing people with wild animal interactions but without real animals present. The content is examined to understand ”how the animals are imagined, experienced and given significance” [37] (p. 2) to determine whether these two cases of virtual animal encounters are viable alternatives, or complementary additions, to in situ wildlife tourism. Further, can these virtual animal encounters assist in the quest to sustain and conserve, and to improve human interactions with animals and nature [38]? For this study, *Shark Dive* and *Hologram Zoo* were attended on 27 September 2023 in the Queensland capital city of Brisbane, which is 75 kilometres north of the coastal tourism hotspot, the Gold Coast, Australia.

### 2.1. Participant Observation

Participant Observation is a qualitative research method aimed at generating and interpreting data with the view to “understanding the nature of phenomena” [39] (p. 13), so is often applied in the form of a case study [40]. As an ethnographic fieldwork method [41], Participant Observation allows researchers to directly observe an event while it occurs [42] and “produce rich descriptive material” [43] (p. 91). Depending upon the objectives of the research and the conditions of the activity under investigation, the style of participant observation can range from “complete participant” to “complete observer” and the varying levels that lie between [44] (pp. 42–44). “At all times the participant observer must retain a high level of introspection to record the event and the researcher’s engagement in it” [22] (p. 1693). There is much to be observed and noted with any phenomena, so a pre-selected theoretical framework helps to narrow the focus of inquiry and influence what the researcher observes and records [39]. Once participant observation has concluded and notes have been taken, “the goal of analysis is to develop a well-supported argument that adds to the understanding of the phenomenon, whether the understanding is phrased in descriptive, interpretative, or explanatory terms” [39] (p. 157). In the case of this study, the researchers are audience members actively participating in *Shark Dive* and *Hologram Zoo*, applying an Animal Studies theoretical framework to observation and analysis.

### 2.2. Animal Studies

Animal Studies is an interdisciplinary research field that considers animals seriously as subjects of investigation [37,38,45] and explores the ways animals intersect with human cultures [45]. This is an important counter to anthropocentrism and its human-centredness [46] because as “more scholars, artists, and activists become interested in concerns ‘beyond the human, animals—their lives, representation, questions of their presence, ethical treatment, political significance and cultural construction—will continue to be brought to the fore” [47] (p. 4). Animal Studies necessitates critical thinking, human responsibility and leadership [38] aspiring to “better think alongside animals” [47] (p. 2), heightening “ethical attention to human–animal relationships” [26] (p. 5), and providing a vision at the level of daily life to inform and revise public policy and human interaction with animals [38].

### 2.3. Data Gathering and Analysis

The application of an Animal Studies theoretical framework provides a critical lens to consider the ways animals are “constructed, represented, understood and misunderstood” [26] (p. 5). Therefore, this study observes, describes, and analyses *Shark Dive* and *Hologram Zoo* by applying the following observation criteria: do the depictions of the virtual animals seem how the animals appear in real life; do the depictions display how the animals may behave in real-life settings; do the depictions of natural environments reflect those that would be home to the animals in real life; do the depictions give insight into the ways humans understand and interact with animals; do the depictions reflect a respectful relationship between humans and animals; do the information sources generate knowledge about, and interest in, the animals; and do the depictions and information encourage conservation awareness and an improvement in the way humans interact with animals and with nature?

“Qualitative research depends on subjectivity…(and) qualitative researchers engage in reflexivity to account for *how* subjectivity shapes their inquiry” [48] (p. 241). Reflexivity provides rigor to qualitative research [48] and is a process “through which we as researchers examine our backgrounds, values, and perspectives shaping our ways of seeing, designing, and participating in research” [49] (p. 404). The two researchers attending *Shark Dive* and *Hologram Zoo* are experienced theatre and communication academics with professional expertise in performing arts management and publicity. Both are passionate about animal welfare and the protection of the natural environment. Neither has cage-dived with sharks, but both have visited zoos and directly experienced wildlife in nature settings within Australia, New Zealand, the Galapagos Islands, and Antarctica. The titles of the two virtual animal encounters attracted the researchers, who were interested in understanding if, and how, the encounters fit within the wildlife tourism industry.

Together, the two researchers attended *Shark Dive* and *Hologram Zoo* in person, each participating with an open mind to be receptive to the experiences. They documented their observations during the events and directly afterward with written field notes. They took photographs of the puppets and stage set at *Shark Dive* to call upon later as visual references. While in the holographic tunnel at *Hologram Zoo* they recorded what they were seeing by speaking into an audio recorder. They then shared their observations with each other to benchmark their reactions against the other. In this discussion, they reflected on their subjectivities as researchers, their previous experiences of animal tourism, and their knowledge and deficits of knowledge that may have impacted their reception. Then, returning to the Animal Studies informed criteria, they sorted through the observational data, looking for commonalities and anomalies, to form their analysis and presentation of results and conclusions.

### 2.4. Shark Dive and Hologram Zoo

*Shark Dive* by Erth Visual & Physical Inc. is a live Australian puppetry production that was presented in Brisbane in 2023 as part of the annual multi-arts Brisbane Festival. Based in Sydney and running for more than 30 years, Erth creates theatrical puppetry experiences inspired by a strong interest in natural history, First Nation stories, sociology, and urban mythology [50]. Audiences attended *Shark Dive* at the Brisbane Circus Centre in Hamilton, 15 kilometres from the city’s commercial business district (CBD), from 17–29 September 2023. The 40-minute-long theatrical performances presented multiple times each day, with tickets priced at AUD 29 per person. *Shark Dive* was promoted as a family-friendly, immersive, and interactive experience suitable for children aged five years and older.

“Ever wanted to dive with sharks but been too afraid? Don’t be! Come close to the Great White without getting wet at a new shark dive experience, created by world-renowned puppeteers, Erth. Plunge into the world of sharks and choose your own audio adventure as you dive below the surface and come face-to-face with these majestic creatures.” [51].

*Hologram Zoo* by Axiom Holographics is an Australian-made augmented reality zoo situated at the Cannon Hill Shopping Centre, 10 kilometres from the Brisbane CBD. Lasers project three-dimensional images in a 20-metre-long tunnel, making objects (plants and animals) appear in the air and look real. The proprietary depth technology used was developed by Axiom Holographics and was voted one of the Best Inventions of 2023 by *Time Magazine* [52]. Promoted as “the world’s most futuristic giant indoor animal theme park” [53], *Hologram Zoo* offers 90 minutes of family entertainment suitable for children of all ages. Tickets are priced at AUD 27–39 per person. The holographic zoo opened in December 2022 as a temporary fixture coinciding with the Christmas school holidays [54]. Intended to close by the end of January 2023, *Hologram Zoo*’s popularity has seen it continue to operate at the shopping centre on a daily basis. Its website indicates that Brisbane has been chosen for the trial of this new technology with the view to establishing more hologram zoos around the world in the future.

“A Hologram Zoo is like a normal zoo. You see elephants, polar bears, kangaroos, and other animals up close and personal. All the animals look real and solid, but they are actually made out of laser light. They are 3D, so you can walk around them and touch them (but your hand goes straight through them as they are made of light).” [53].

The Chief Executive Officer of Axiom Holographics, Bruce Dell, states that Hologram Zoo “…allows people to see animals that they may not otherwise have the opportunity to encounter…when a giant whale swims past you, everyone goes into a reverence” [55]. This affordance of seeing animals they might not otherwise see, correlates with the role of traditional zoos and aquaria as discussed above.

Both arts and design based, these virtual animal entertainments provide safe opportunities to be near wild animals. Close encounters with wild animals can be dangerous to humans [7], but these virtual forms of human–animal interaction are perfectly safe. Proximity to the animals is an influential aspect in the marketing of the activities because this meets the desires of those who engage in wildlife tourism, which provides opportunities for close encounters [32,56].

## 3. Results

In line with a Participant Observation approach, the following descriptions detail the results of the researchers’ experiences of *Shark Dive* and *Hologram Zoo*. This description leads to analysis considering how the animals have been imagined, experienced, and given significance to determine whether their representation aligns with wildlife tourism’s endeavours to sustain and conserve nature and to improve human interactions with animals.

### 3.1. Shark Dive

#### 3.1.1. Act One

Up to 25 patrons per session quietly enter a darkened, rectangular room with bench seats running along the length of the walls on either side. Fifteen people, mostly children, attend our session and as we sit facing each other, we are informed that this is the decompression chamber to prepare us for our soon-to-be-experienced shark dive. A decompression chamber would not be used for a real shark cage-dive, but this fictional adjustment stands in for boat travel and scuba diving gear tourists would wear. Our guide leads us through slow and deep breathing exercises to lower our heart rates and to create a sense of calm. We are each given a cold, wet cloth to place over our faces to ready us for the imaginary ocean we will soon be immersed in. Hand signals are demonstrated so that we can communicate without speaking once in the surface cage, and we are also told not to stick our hands or legs out through the bars of the cage. Finally, we are given the option of ‘taking a risk’ or ‘playing it safe’ to determine which set of headphones we will wear during the shark encounter. Those playing it safe hear calming ‘new age’ instrumental music, while those taking a risk hear more suspenseful, upbeat instrumental sounds. No information is given about sharks as part of this preparation scene.

#### 3.1.2. Act Two

We enter the next performance space by being helped through a submarine-like doorway or hatch to find ourselves in a large and high-ceilinged room, with deep blue light filling the dimly lit environment revealing a cage at its centre. The blue lighting signifies the deep water of the ocean and denotes calmness and beauty. The cage design is reminiscent of the surface cages used in real shark dives off the coast of southern Australia, which creates a sense of realism within this fictional world. Participating in the fiction, we are encouraged to move our arms as if swimming towards the cage. We all step inside the cage, which is then locked, and we await the Great White shark. Puppeteers dressed in black emerge, manipulating life-sized and life-like puppets of stingrays, leafy seahorses, a school of fish, and a couple of grey nurse sharks. Each is realistically portrayed, and immediately, many of the children in the cage are compelled to thrust their arms out to touch the puppets as they glide past. All the while, in the cage, the music plays in our headphones, accompanied by the whispered voice of a female narrator speaking as if she is the shark we are waiting to see. She gives some insight into sharks; their characteristics, physical attributes, and lives in the ocean. Just prior to the Great White shark appearing, the narrator says, “Respect me and I will respect you”.

The large, life-like puppet of the Great White is controlled by four puppeteers. She swims around the cage a few times, coming up close to the enclosure, her eyes seemingly looking straight at us. Noticeably, the children refrain from placing their hands outside the cage this time. Over the sound of the music in the headphones, some of the children can be heard excitedly shouting to each other and gesturing toward the shark. It seems they are thrilled to see the shark, just as those who participate in real shark cage-dives are. The Great White lifts up over the top of the cage and then returns to circling. Without warning, she lurches at the bars with her mouth wide open with jagged teeth exposed. The shark’s swimming motion, body size, shape and colour, and teeth when exposed, all appear to be natural. Non-flash photography is permitted and the photographs we take during the cage-dive are later found to be very similar to photographs posted online from those participating in real shark dives. Throughout the encounter the narrator through our headphones speaks positively about sharks, leaving a lasting impression that they are magnificent animals who are beautiful and to be respected. After a time, the shark swims away from the cage and out of sight behind a black curtain. We are then all ushered out of the cage, and process through another submarine-like doorway or hatch to a room configured similarly to the first. This time, the two rows of bench seats prompt the audience to look in the one direction—toward the cage we just left.

#### 3.1.3. Act Three

We look through a sheer black curtain, which enables us to see the next wave of patrons enter the surface cage (approximately 10 metres from us) and to see the puppeteers in black manipulating the same puppets as earlier to circumnavigate the cage. As we engage with the scene, this time as observers rather than as active participants, the music in our headphones is uplifting and inspirational. The female narrator’s voice continues but is now speaking as a human rather than as a shark. As the Great White enters the performance space again, the narrator points out some of the similarities between sharks and humans. This aids in building a connection between humans and sharks, and potentially induces empathy when she reminds us that by entering the ocean, we have entered the shark’s world and that we, as a species, are more dangerous to sharks than they are to us. The narrator explains we can flee the ocean to dry land to escape the shark, but the shark has nowhere to flee beyond the ocean when humans attack.

The combination of listening to the inspirational music and the female voice, and watching others go through what we just went through brings a feeling of content detachment and reflection. The people in the cage look alien in the landscape, held inside a metal cage within a deep ocean. This was us only moments before, but now we gain a sense of what the shark and other marine animals see when swimming by the cage, and we wonder what they must be thinking about us. This contemplation and relational thinking make us receptive to the narrator’s suggestion that cage-dives may not be allowed much longer because we disrupt sharks’ lives with such activity, and we change their behaviours as a result. As the shark puppet disappears behind her curtain, a door at the end of the room we are in opens and we make our way out, returning to the natural light of the foyer as our experience of *Shark Dive* concludes.

#### 3.1.4. Analysis

The animals in *Shark Dive* are represented by puppets manipulated by human puppeteers. The puppets appear to be anatomically correct for the animals they portray, and their movements and interactions with us seem naturalistic. The animal puppets/characters are standing in for real animals in this performance rather than symbolically representing humans or human priorities. The narration acts as a form of onsite interpretation. During our time in the cage, the narration gives voice to sharks as the information shared is as if from a shark’s perspective. The content is positive and educative, giving details about sharks and their lives in the ocean. When in the observation room following our time in the cage, we are encouraged to reflect upon and consider our relationships as humans with sharks. The narrator explains the similarities we share with sharks and the impact we have on their lives when we enter the ocean. While listening to the narration, we observe the animals circling the cage filled with other humans. This provides an opportunity to view humans as the animals might, placing ourselves in their position, which engenders empathy and critical reflection upon the role we played while in the cage. *Shark Dive* provides a positive representation of marine life, particularly of the Great White shark. It educates the audience about sharks and about our relations with them. It encourages self-reflection, generates awareness about the need for conservation, and promotes sharks as beautiful and magnificent animals.

### 3.2. Hologram Zoo

Upon arrival, we are asked to select a region (Australia, Africa, Arctic, or World of dinosaurs) whose animals and landscapes will feature in the holographic tunnel. We choose Australia, as this is where we reside and have travelled quite extensively, so comparisons between the virtual environments and our memories of real encounters can be made. We are then asked to select one interactive animal experience from Coral Reef; Open Ocean; Dinosaurs on land; and Dinosaurs in the sky and sea. We choose the open ocean, again as an environment with which we are familiar.

#### 3.2.1. Phase One—Onsite Interpretation

The onsite interpretation’s content varies depending upon the geographic location chosen to experience. We are seated to engage with a 10-minute lecture delivered by a digitally animated female figure who outlines the characteristics and uniqueness of Australian marsupials, monotremes, and the cassowary. The data she provides is appropriate for children to comprehend and her informative narration is accompanied by animated images of the animals to view. This frames the visit as zoos would by providing educational information to introduce the animals onsite. We make our way to the holographic tunnel to witness Australian animals and landscapes.

#### 3.2.2. Phase Two—Australian Tunnel and Arctic Tunnel

We put on 3D spectacles and enter the 20-metre-long tunnel, where we are prompted by a horizontal moving green light to slowly walk along, looking at the images projected onto one side of the space. We move through and exit the tunnel, returning to the start and walking through the tunnel again, in total, five times. Each time, we are faced with a different Australian natural landscape (e.g., beach or eucalypt forest) and some of the animals that would typically reside there (e.g., seabirds or koalas). A 3D effect is achieved, which makes the scenes appear realistic, with animals interacting with their environment through actions such as swimming, flying, sitting in trees, and eating. The viewpoint we hold is as if standing in the featured environment watching on, with animals at times coming closer toward us for a better look or even jumping over our heads. In one scene, set in a gorge with a freshwater creek running through it, we are positioned as if in the creek with the surface of the water at eye height. We can see through the water to the animals swimming in it as well as above the water to those walking on land. We observe a platypus enter the water and swim nearby, along with a very large estuarine crocodile whose natural movements are convincingly presented. The crocodile turns to look at us and then lurches forward in attack, realistic enough to make us both jump back without thinking. Virtual water spray flows over us as the crocodile backs off.

Although named *Hologram Zoo*, experiences such as those in the Australia-themed tunnel are more reminiscent of those situated in nature rather than behind the walled or fenced enclosures of zoos. The depiction of the landscapes resonate strongly with real landscapes we have experienced, as do the majority of the animals we see in the tunnel. The kangaroos appear life-like, although we note some are identical, as if the representations are based on one kangaroo. The movement of the kangaroos also stands out as not quite right. As we turn our attention to them hopping, we recognise that their legs and tails move as expected, but their torsos are stiff and not angled as we had observed in real-life situations. This makes them look slightly unusual. Someone who is not familiar with kangaroos, however, will not have necessarily noticed this. Inside the tunnel, the experience is immersive. There is no accompanying onsite interpretation to provide information about the many animals we see. However, by observing the configuration of the animals, their movements, and their placement within particular environments, we are given visual clues to gain insight into their lives.

We also view the Arctic display in the same way as the Australian display—we take five stints through the tunnel, with each occasion presenting a different landscape and different animals. In the holographic tunnel, cold air blows out at us from the direction of the images to add to the feeling of being in a cold environment. Projected snowflakes fall realistically around the observer. Yet, overall, the Arctic experience does not seem as accurate as the one prior, and as such, is not as satisfying. There are Arctic and Antarctic animals together in the same environment (e.g., a walrus and penguins), which reduces credibility. There are also a few different types of penguin depicted, but it seems like their image had been drawn from virtual penguins rather than from real animals. As we have been to Antarctica and viewed four different types of penguin up close while there, those virtually depicted are quickly identified as not quite appearing nor moving naturally. The other interesting difference to the Australian experience is the feeling of looking through glass as in an aquarium rather than being situated in the scenes. This is apparent when encountering cetaceans swimming by, particularly when a large orca swims directly toward us, only halted by the virtual, transparent barrier that cracks upon impact like glass. Unlike in response to the crocodile lunge, neither of us moves, perhaps subconsciously aware of the virtual glass barrier to protect us.

#### 3.2.3. Phase Three—Interactive Ocean Experience

We enter a small, enclosed space housing five chairs, which face toward the other end of the room where the holographic images are on display. Six short, interactive scenarios are presented, which require one person at a time to participate. Wearing special interactive glasses and holding a ‘wand’, we see the animals in 3D moving in the space for us to physically respond to. For instance, in the first scene, flying fish are depicted landing in a rowboat that the participant looks to be standing in. Using the wand, which appears as a holographic shovel, the fish are scooped out of the boat and returned to the ocean. As the scene progresses, more and more fish fly into the boat and need to be saved. As each scene plays, we can hear a recorded male voice telling us a little about the animals featured and prompting us to interact in certain ways with them.

The troubling aspect of the interactive ocean experience is the form of interaction we are prompted to make in some scenes. With the exception of two scenes, our interaction involves using the wand to push away, swing at, shoot at, or fend off many of the animals we encounter. For example, in the third scenario, we see turtle hatchlings dig their way out of the sand to then make their way to the ocean. However, their attempts can be thwarted by predatory seagulls who swoop down upon them. The male narrator instructs the participant to shoot at the seagulls with a non-lethal sandgun to protect the turtles. More and more seagulls arrive on the scene, prompting the gun holder to use the weapon like a semi-automatic rifle, blasting away at the birds in all directions. The birds are not shown to die or to fall from the sky injured, however, they do lose some feathers and turn to fly away. Although defending the baby turtles, it does not feel appropriate to shoot at another animal species that is exhibiting natural behaviours to seek food. Other scenes are similar, such as hitting a Great White shark repeatedly with an oar as s/he lurches forward to attack the participant standing in an old wooden ship; scooping out the flying fish can soon turn to swinging at them as if with a baseball bat due to the sheer numbers encroaching; and shocking a giant squid with electric current to prevent the squid from completely damaging a submersible which houses the participant.

Having attended *Shark Dive* less than two hours prior to *Hologram Zoo*, we are struck by the opposing depictions of the sharks in the virtual animal encounters. The two times sharks are represented at *Hologram Zoo*, both during the interactive open water experience, they are portrayed as aggressive animals who seek to attack the participants. The participants are prompted to hit out at the sharks in self-defence. This portrayal of sharks is in keeping with popular stereotypes, which present sharks as “killing machines” and “man eaters”, reinforcing fear and negative connotations [11,57]. This is significantly different from the *Shark Dive* performance, where we are encouraged to view them as beautiful animals who hunt for food when hungry and who only encounter humans when we are placed directly in their watery environment.

#### 3.2.4. Phase Four—Bridge Animal Experience

The final holographic experience entails walking over a bridge within a large, high-ceilinged room to witness animals and landscapes projected onto the floor and walls. We walk up and over the bridge five times, with each occasion seeing different 3D images. Those coming out from the walls are large projections isolated from any natural environment (e.g., the head and neck of a giraffe). This gives the impression of an art gallery installation rather than a zoo atmosphere. When standing looking over the bridge to the images projected on the floor, it is like looking down into a variety of environments including a ravine where animals are viewed amongst nature. Again, at times, we encounter animals together in one type of environment when this does not naturally occur (e.g., gorillas and cassowaries). No onsite interpretation is provided.

#### 3.2.5. Analysis

The animals and landscapes in *Hologram Zoo* are presented in 3D via augmented reality technology. For the most part, the 3D images appear to be anatomically correct for the animals they portray, and their movements and interactions seem naturalistic. However, the accuracy of representation is questioned at times when observing the kangaroos hopping and the penguins moving about on the ice. The authenticity of the representation is also diminished when we are faced with a walrus and penguins together in the one environment, along with cassowaries and gorillas sharing the same forest. Mostly, the animal characters in *Hologram Zoo* are standing in for real animals rather than symbolically representing humans or human priorities. Yet, in some instances, the depiction of the animals is skewed to reflect human bias based on myth and stereotypes. For example, we do not see Great White sharks swimming around, foraging, or even seizing upon marine prey at *Hologram Zoo*. Instead, the Great White shark is aggressive and on the attack. This encounter is a popular construction of the Great White, fuelled by *Jaws* [57] and other films since rather than a representation of a shark in the wild.

In some of the interactive scenarios, we are prompted by the recorded male voice and by the conditions of the game to be necessarily violent to some of the virtual animals. These constructed scenarios pitch humans against animals and encourage negative associations with those animals. While non-fatal, the interactive moments draw upon popular tropes of gaming where actions like shooting are common practice [30] and, thus, likely a familiar activity to families participating. But there could be other ways of interacting with the virtual animals that do not include such negativity and violence, potential conditioning influences for future behaviours in the natural world. Onsite interpretation provides some insight about the animals during the interactive scenarios from the recorded comments from the male narrator, along with the information session at the very start hosted by an animated female human character. The information shared at *Hologram Zoo* explains a little about the uniqueness of certain animals (such as monotremes) but does not include details about their conservation status or the ways in which humans impact their lives or could assist in improving their lives. There is no information provided about many of the other animals we encounter in the tunnel or on the bridge. Our insights are mostly derived from visually observing the holograms to see the animals interacting with their environments. This is useful, with the exception of cases as described above where the depiction is inaccurate and/or reflects an anthropocentric viewpoint. *Hologram Zoo* does not appear to have a pervading conservation education agenda on display, as accredited zoos and aquaria do. It does not keep animals captive and, therefore, does not stifle wild animals’ agency as living beings, but it does represent some of the animals in ways that reinforce negative stereotypes and encourage humans to be aggressive toward them. This experience presents some animals as ‘the enemy’ and puts forward violence as a defensive solution, potentially teaching people (particularly children) to be disrespectful and hurtful to certain species.

## 4. Conclusions

When addressing this study’s first question, both virtual encounters can be understood to be viable alternatives, or at the very least, viable complementary additions to wildlife tourism offerings delivered in nature. When addressing the second research question, this study confirms *Shark Dive* does aid conservation awareness and contributes to the improvement of human interactions with animals and nature. It employs a form of interpretation [15] common to wildlife tourism via the narrator, who speaks to us throughout the shark encounter about sharks and human relations with them. The narrator prompts us to consider human impact upon sharks and the viability of shark cage-diving in the future. There is time within *Shark Dive* to self-reflect [3], which is another component of successful interpretation, generating understanding and advocacy for nature. Therefore, this paper argues that *Shark Dive* reinforces sustainable wildlife tourism ideals through its virtual presentation of animals. *Hologram Zoo*’s 3D content showcases a wide variety of animals introducing people to animals they may have never experienced in real life [16]. This can induce greater interest in the animal kingdom. The virtual experience is emotionally engaging and provides learning opportunities through observation and participation. The holographic tunnel demonstrates how effectively augmented reality technology can deliver a virtual wildlife experience. However, the species information provided is limited, and there is no mention of conservation. The examples of violent interactive gaming sessions with the animals do not advocate for responsible interactions with wildlife, so they do not improve human interactions with animals and nature. Thus, we argue that, at present, *Hologram Zoo* is impressive, but does not consistently reinforce sustainable wildlife tourism ideals through its virtual presentation of animals.

### Recommendations

*Shark Dive* and *Hologram Zoo* have been shown to be affordable and entertaining, providing an enjoyable hour or two engaging with virtual representations of wild animals. Both experiences are successful in meeting the needs of families who wish to encounter active, wild animals up close in a safe environment. *Shark Dive* provides an alternative narrative about the Great White Shark to counteract negative stereotyping and the human fears associated with it. Interestingly, it is a virtual animal encounter that draws inspiration from real white shark cage-diving, yet it highlights for the audience the criticism of this activity’s impact on marine life. *Hologram Zoo* demonstrates how a virtual animal encounter can entertain and educate. As the scope of geographic regions and the variety of animals and environments that will be represented grows, there is an opportunity to refine the ways in which nature is portrayed and the forms of interaction encouraged between humans and animals. The technology provides convincing 3D animal images and introduces children and adults to a range of flora and fauna that they may not have encountered previously, and are guaranteed to see. This introduction can encourage greater interest in, and understanding of, the real animals who are represented. *Hologram Zoo* is housed in a shopping centre that is readily accessible and affordable, a familiar venue for families, and easy to replicate elsewhere. With a reconfiguring of the augmented reality experience to incorporate an enhanced pro-environmental stance and respectful human-animal interactions in all of the gaming sessions *Hologram Zoo* could exemplify the ideals of sustainable wildlife tourism.

As virtual wildlife experiences, both of these entertainments highlight the potential for greater use of a range of virtual possibilities in showcasing nature, thus promoting awareness and connection to issues of the natural world. Particularly in locations where people may have reduced opportunity to view the animals depicted or where urban residents may avail themselves of the virtual experience as part of their busy week, entertainments such as these could serve to place the natural world at the forefront of people’s minds. Positive and emotionally engaging depictions of nature serve to remind people to reflect upon the natural world and our relationship to it. In this way, virtual animal encounters hold the potential to be both engaging and educative in a similar manner to traditional wildlife tourism but afford ease of access and avoid potential negative impacts of human and wild animal interactions.

## Data Availability

The data presented in this study are available in article.
